# Supplementary material for: Enhanced Antitumoral Activity of Encapsulated BET Inhibitors When Combined with PARP Inhibitors for the Treatment of Triple-Negative Breast and Ovarian Cancers
Source: Cancers (Basel). 2022 Sep 15;14(18):4474. doi: 10.3390/cancers14184474 (PMC9496913; doi:10.3390/cancers14184474)
Supplement: Supplementary file 1 [file cancers-14-04474-s001.zip › cancers-1869351-supplementary.pdf]

Electronic Supporting Information

Enhanced antitumoral activity when combining PARP inhibitors with encapsulated BET inhibitors for the treatment of BRCA-mutated cancers

Alberto Juan,[1#] María del Mar Noblejas-López,[1,2#] Iván Bravo,[1,2] María Arenas-Moreira,[1,2] Cristina Blasco-Navarro,[2] Pilar Clemente-Casares,[1,2] Agustín Lara-Sanchez,[3] Atanasio Pandiella,[4] Carlos Alonso-Moreno[1,2]* and Alberto Ocaña[5]*

[1]Centro Regional de Investigaciones Biomédicas (CRIB), UCLM, Albacete-02008, Spain

[2]Translational Oncology Laboratory, Translational Research Unit, Albacete University Hospital, Albacete-02008, Spain

[3]Universidad de Castilla-La Mancha. Facultad de Farmacia de Albacete, Albacete-02008, Spain

[4]Universidad de Castilla-La Mancha. Facultad de Ciencias y Tecnologías Químicas, Ciudad Real-13005, Spain

[5]Centro de Investigación del Cáncer-CSIC. IBSAL- Salamanca and CIBERONC, Salamanca-37007, Spain

[6]Experimental Therapeutics Unit, Hospital clínico San Carlos, IdISSC and CIBERONC, Madrid-28040, Spain

[#]Both authors contributed equally

(*) corresponding authors:

Carlos Alonso-Moreno, PhD. Unidad nanoCRIB, Centro Regional de Investigaciones Biomédicas, Universidad de Castilla-La Mancha, 02008 Albacete, Spain. E-mail: Carlos.amoreno@uclm.es. Phone number: +34967599200

Alberto Ocaña, PhD. Experimental Therapeutics Unit, Hospital clínico San Carlos, IdISSC and CIBERONC, Madrid, Spain. Email: alberto.ocana@salud.madrid.org. Phone number: +34 635681806.

.

A

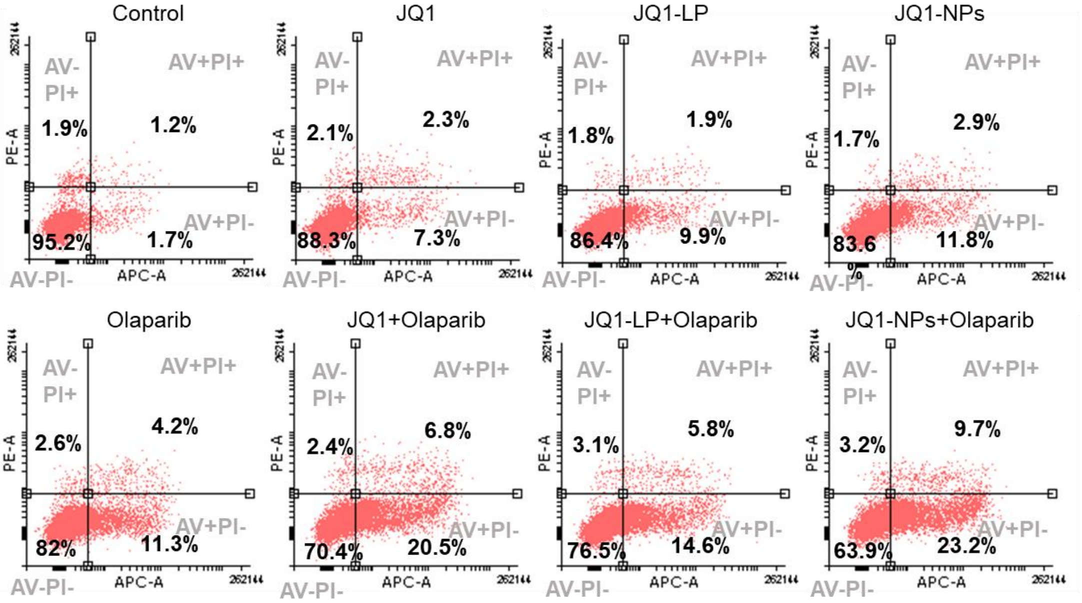

B

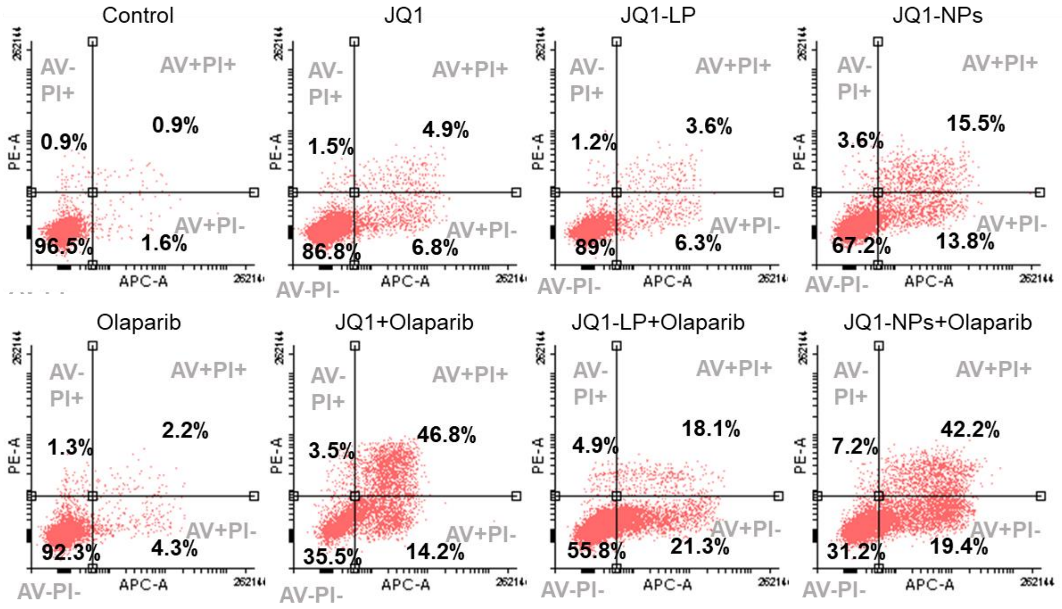

Figure S1. A. JQ1 free and JQ1-encapsulated forms synergize with olaparib in breast cancer cells MDA-MB-231. Dot plot represent percentage of cells Annexin V positive/negative and Propidium Iodide positive/negative after each treatment (400 nM). * $p \leq 0.05$; ** $p \leq 0.01$ and *** $p \leq 0.001$. **B.** JQ1 free and JQ1-encapsulated forms synergize with olaparib in ovarian cancer cells OVCAR8. Dot plot represent percentage of cells Annexin V positive/negative and Propidium Iodide positive/negative after each treatment (400 nM). * $p \leq 0.05$; ** $p \leq 0.01$ and *** $p \leq 0.001$.
